# Real-World Insights: End-of-Life Care for Patients with Terminal Gastric Cancer at Home

**DOI:** 10.1089/pmr.2024.0104

**Published:** 2025-04-02

**Authors:** Hiroaki Yamane, Aki Yoshimitsu, Etsushi Akimoto, Tomoyuki Abe, Motoi Yamane

**Affiliations:** ^1^ Department of Surgery, Yamane Clinic, Hiroshima, Japan.; ^2^ Department of Clinical Oncology, JA Hiroshima General Hospital, Hiroshima, Japan.; ^3^ Department of Surgery, Akimoto Clinic, Hiroshima, Japan.; ^4^ Department of Gastroenterological Surgery, National Hospital Organization Higashihiroshima Medical Center, Hiroshima, Japan.

**Keywords:** end-of-life care at home, gastric cancer, prognosis

## Abstract

**Background:**

As the Japanese population continues to age, home health care provision has greater significance. However, the number of patients with terminal cancer receiving end-of-life care at home remains limited, and predicting the prognosis of patients with terminal-stage gastric cancer is challenging.

**Objective:**

To analyze the real-world data of patients with terminal gastric cancer receiving end-of-life care at home to provide insights into their care and health outcomes.

**Design:**

A two-center retrospective study.

**Setting/Subjects:**

This study focused on terminal patients with gastric cancer who died at home between 2021 and 2024 in Japan. A total of 27 participants (14 males and 13 females) were included in the study, with a median age of 78 years.

**Measurements:**

First, we analyzed the detailed clinical course of the patients during home care. Second, we performed a comparative analysis by dividing the patients into two groups based on median overall survival (OS).

**Results:**

The median OS during home care was 22 days. The patients were divided into two groups: long OS (OS-L) and short OS (OS-S). Strong opioid use and home oxygen therapy were recorded in 22 and nine patients, respectively. In the OS-S group, oral intake was significantly reduced (25.0% vs. 66.7%, *p* = 0.032). There was a notable difference in serum albumin levels between the two groups (2.8 vs. 2.4 mg/dL, *p* = 0.038). The neutrophil-to-lymphocyte ratio (NLR)/albumin was significantly higher in the OS-S group compared to the OS-L group (1.0 vs. 2.3, *p* = 0.032).

**Conclusions:**

Oral intake, serum albumin level, and NLR/albumin were important prognostic factors in end-of-life care at home of patients with terminal gastric cancer.

## Key Message

The prognosis for patients with terminal gastric cancer at home remains unclear. In this study, we performed a retrospective search for prognostic factors in patients with terminal gastric cancer treated at home. The results showed that patients with decreased oral intake and low serum albumin had a significantly deteriorated prognosis.

## Introduction

Home-based end-of-life care is pivotal in enhancing patient outcomes and alleviating the burden on the health care system. A body of evidence demonstrates that home visits reduce the need for emergency room visits and provide vital support to patients, particularly those who are unable to access hospitals.^1^ This approach encompasses symptom management, emotional support, and minor medical procedures. In addition, it is associated with enhanced perceptions of care among family members and the wider community. During the pandemic, home-based palliative care played a pivotal role in maintaining continuity of care, psychosocial support, and end-of-life care.^2^ Home care for individuals diagnosed with terminal gastric cancer lies in its capacity to provide a comfortable and familiar environment during the final days of life.

Gastric cancer is a common malignancy in Japan,^3^ and various prognostic factors have been identified to date.^1,4,5^ However, most existing studies focus on the prognosis of end-stage gastric cancer or prediction of postoperative outcome of gastric cancer.^1,4,5^ Thus, prognosis of patients with terminal gastric cancer who receive end-of-life care at home remains underexplored. The lack of prediction prognosis is known to cause anxiety in family members.^6,3^ Furthermore, family members struggle with the emotional and physical demands of caregiving, which can affect the quality of care provided. Although models such as the Palliative Prognostic Index (PPI), the neutrophil-to-lymphocyte ratio (NLR), Prognostic Nutritional Index (PNI), and NLR/albumin (Alb) provide some guidance,^1,5,7,8^ the accurate prediction of outcomes remains challenging, particularly in home care settings. Home care involves the collaboration between medical providers and families, underscoring the need for simple and reliable prognostic tools; however, evidence of prognostic prediction in patients with terminal cancer receiving end-of-life care in home settings remains limited.

This study aimed to address this gap by focusing on survival-related factors in patients with terminal gastric cancer receiving end-of-life care at home. Understanding these factors may lead to better patient management and end-of-life care.

## Patients and Methods

### Patients

In this two-center retrospective study, we enrolled patients with terminal gastric cancer who remained at home between January 2021 and June 2024 in Japan. Patient data were collected from the electronic medical records. The inclusion criteria were as follows: diagnosis of terminal gastric cancer, termination of or did not pursue active treatment (surgery, chemotherapy, and radiation), age older than 18 years, and being end-of-life cared for at home or in serviced housing for older people.^9^ Staging was performed according to the 8th Union for International Cancer Control guidelines.^10^ The exclusion criteria included transfer to palliative care units or hospitals.

Japan’s medical insurance system, established in 1961, provides health coverage for all citizens through national and social insurance schemes. Home care is also provided under the medical insurance system, and patients who have difficulty going to the hospital can receive regular home care at home or at a facility if they wish.

Regarding the analysis of data in this study, the individual consent of the participants was waived by using the opt-out method. Approval for this study was obtained from the Medical Governance Research Institute (MG2024-02). All methods were performed in accordance with the relevant guidelines and regulations.

### Treatment

Patients were treated based on their symptoms, including the administration of strong opioids and the initiation of home oxygen therapy (HOT) as needed. Chemotherapy and radiotherapy were not provided in home care settings. The focus of treatment was on end-of-life care and symptom management, addressing the needs of patients with terminal gastric cancer without the involvement of active oncology treatments from the hospital.

### Evaluation and statistical analysis

First, we analyzed the detailed clinical course of the patients with terminal gastric cancer receiving end-of-life care during home care, including their condition at the time of visit, comorbidities, cancer stage, strong opioid and HOT use, and blood examination data. Oral intake was classified into two categories: absent and moderately reduced or severely reduced. Blood samples were collected 1 week before and after initiating home care. To differentiate between short- and long-term home care cases, we performed a comparative analysis by dividing the patients into two groups based on the median survival time. The parameters included age, sex, stage, strong opioid use, HOT use, and blood examination results. NLR was defined as the neutrophils count multiplied by the lymphocytes count^7^; NLR/Alb was defined as NLR by Alb (mg/dL).^5^ PNI was calculated from the formula 10 × Alb (mg/dL) + 0.005 × lymphocyte count.^8^ Statistical analyses were performed using *U* tests for continuous variables and Pearson’s tests for nominal variables, with significance set at *p* < 0.05. Sensitivity, specificity, and the area under the curve (AUC) for overall survival (OS) were determined using receiver operator characteristic analysis. JMP version 15 (SAS Institute, Cary, NC, USA) was used for calculations.

## Results

### Patient background and blood examination

The characteristics of the 27 consecutive patients enrolled in this study are presented in Table 1. The median age of the patients was 78 (43–100) years. Of the 27 patients, 14 were male (51.9%) and 13 were female (48.1%). The median OS was 22 days after palliative treatment. Patients were divided into two groups based on the OS: 12 patients comprised the long-term OS group with survival of more than 23 days (OS-L; 44.4%), and 15 patients comprised the short-term OS group with survival of 22 days or less (OS-S; 55.6%). Half of the patients had a severely reduced oral intake (48.2%). In 22 cases, the reason for starting home care was the progression of the disease (81.5%). The reasons for home care in the other five patients were related to advanced age and dementia (18.5%). Strong opioids were used in 22 cases (81.5%), and in five of these cases (18.5%), treatment with continuous subcutaneous injection of strong opioid commission was used. HOT was administered in nine cases (33.3%); eight of these cases had HOT introduced after home care was initiated (29.6%).

**Table 1.** Background Information of Patients with Terminal Gastric Cancer

Median age (min–max), years	78 (43–100)
Male/female sex	14 (51.9%)/13 (48.1%)
Comorbidity	
Hypertension	10 (37.0%)
Hyperlipidemia	2 (7.4%)
Diabetes mellitus	2 (7.4%)
Dementia	7 (25.9%)
Place of death	
At home	26 (96.3%)
Serviced housing for older People	1 (3.7%)
Median overall survival at home (min–max), days	22 (0–406)
OS-long (OS-L)	12 (44.5%)
OS-short (OS-S)	15 (55.5%)
Condition at time of visit	
Performance status 1/2/3/4	8 (29.6%)/10 (37.0%)/ 7(25.9%)/2 (7.4%)
Oral intake absence/moderate reduced/severely reduced	7 (25.9%)/7 (25.9%)/13 (48.2%)
Delirium	7 (25.9%)
Primary TNM stage	
1/2/3	8 (29.6%)
4	19 (70.3%)
Recurrence	3 (11.1%)
Histopathological classification	
Differentiated type	5 (18.5%)
Undifferentiated type	7 (25.9%)
ND	15 (55.5%)
Metastasis	
Liver	9 (33.3%)
Lung	3 (11.1%)
Peritoneal dissemination	11 (40.7%)
Strong opioid usage	22 (81.5%)
Opioid type (max dose/day)	
Fentanyl transdermal (6 mg)	16 (59.3%)
Fentanyl buccal (100 mg)	1 (3.7%)
Oxycodone oral (5 mg)	1 (3.7%)
Hydromorphone oral (8 mg)	1 (3.7%)
Morphine suppository (30 mg)	1 (3.7%)
Rescue use only	1 (3.7%)
CSI use only	1 (3.7%)
Opioid CSI	5 (18.5%)
CSI type (max dose/day)	
Oxycodone (84 mg)	3 (11.1%)
Morphine (192 mg)	2 (7.4%)
HOT	9 (33.3%)
Median HOT usage period (min—max), days	13 (2–19)

CSI, continuous subcutaneous injection; OS, overall survival; HOT, home oxygen therapy; ND, not describe; TNM, tumor, node, metastasis.

The blood examination data summarized in Table 2 included some missing data due to the study’s retrospective nature.

**Table 2.** Blood Examination Data for Patients with Terminal Gastric Cancer

	Lack number	Median (min–max)
WBC (/uL)	0	7990 (2300–18,120)
Neutrophil (/uL)	1	5800 (1400–15,840)
Lymphocytes (/uL)	1	1625 (200–17600)
Alb (g/dL)	2	2.6 (1.5–3.9)
Cr (mg/dL)	2	0.76 (0.31–2.71)
T-bil (mg/dL)	1	0.5 (0.3–39)
AST (U/L)	1	26.5 (14–208)
ALT (U/L)	1	18.5 (8–97)
C-reactive protein (mg/dL)	5	2.6 (0.0–34.6)
NLR	1	2.8 (0.3–12.6)
PNI	2	35.5 (20.5–102.0)
NLR/Alb	3	1.5 (0.3–6.7)

Alb, albumin; ALT, alanine aminotransferase; AST, aspartate aminotransferase; Cr, creatinine; NLR, neutrophil-to-lymphocyte ratio; PNI, prognostic nutritional index; T-bil, total bilirubin; WBC, white blood cells.

### Association between OS and patient characteristics

No other significant associations were found between OS and variables such as age, sex, gastric cancer stage, strong opioid use, and HOT (Table 3). The OS-S group tended to have a less significant reduction in oral intake than the OS-L group (25.0% vs. 66.7%, *p* = 0.032). These findings suggest that oral intake may play a role in survival outcomes.

**Table 3.** Comparison of Patient Characteristics in the Overall Survival Groups

	OS-L *n* = 12	OS-S *n* = 15	*p*
Age (years)	84 (43–91)	72 (54–100)	0.070
Sex	Male 5 (18.5%)	Male 9 (33.3%)	0.343
Hypertension	7 (58.3%)	3 (20.0%)	0.040
Hyperlipidemia	2 (16.7%)	0 (0%)	0.100
Diabetes mellitus	1 (8.3%)	1 (6.7%)	0.870
Dementia	5 (41.7%)	2 (13.3%)	0.095
PS ≥ 3	3 (25.0%)	6 (40.0%)	0.411
Oral intake severe reduced	3 (25.0%)	10 (66.7%)	0.032
Delirium	3 (25.0%)	4 (26.7%)	0.922
OS	81 (28–406)	13 (0–22)	<0.001
Stage IV	8 (66.7%)	11 (75.3%)	0.931
Distant metastasis at home care	9 (75.0%)	13 (86.7%)	0.438
Liver metastasis	3 (25.0%)	6 (40.0%)	0.411
Lung metastasis	1 (8.3%)	2 (13.3%)	0.681
Peritoneal dissemination	4 (33.3%)	7 (46.7%)	0.484
Opioid usage	10 (83.3%)	12 (80.0%)	1.000
Opioid CSI	2 (16.7%)	3 (20.0%)	1.000
HOT	4 (33.3%)	5 (33.3%)	1.000

### Association between OS and blood examination

A comparison of OS and blood examination was shown in Table 4. No statistically significant differences were observed between the groups regarding white blood cell count, renal function, or liver function. In addition, C-reactive protein levels, an inflammatory marker, showed no significant differences. Established cancer prognosticators, such as NLR and PNI were also compared, and no significant differences were observed. However, Alb, an indicator of nutritional status, was significantly lower in the OS-S group compared to the OS-L group (2.8 mg/dL vs. 2.4 mg/dL, *p* = 0.038). Additionally, NLR/Alb was significantly higher in the OS-S group compared to the OS-L group (1.0 vs. 2.3, *p* = 0.032). At the cutoff value of less than 2.4 for Alb, Alb yielded an AUC of 0.744, with 54% sensitivity and 62.9% specificity. At the cutoff value of more than 2.2 for NLR/Alb, NLR/Alb yielded an AUC of 0.757, with 58.3% sensitivity and 50.0% specificity.

**Table 4.** Comparison of Blood Examination Results in the Overall Survival Groups

	OS-L *n* = 12	OS-S *n* = 15	*p*
WBC (/uL)	8135 (4500–14300)	7990 (2300–18120)	0.903
Neutrophil (/uL)	5800 (2500–9500)	5832 (1400–15840)	0.700
Lymphocytes (/uL)	1958 (700–5700)	1219 (200–17600)	0.411
Alb (g/dL)	2.8 (2.2–3.9)	2.4 (1.5–3.2)	0.038
Cr (mg/dL)	0.70 (0.31–2.71)	0.92 (0.42–2.64)	0.355
T-bil (mg/dL)	0.4 (0.3–1.4)	0.7 (0.3–39)	0.131
AST (U/L)	25 (14–87)	28 (15–208)	0.520
ALT (U/L)	12 (8–43)	20 (10–97)	0.063
C-reactive protein (mg/dL)	2.1 (0.1–19.7)	3.6 (0–34.6)	0.5310
NLR	2.7 (1.3–12.6)	5.5 (0.3–12.5)	0.440
PNI	36.9 (32.5–56.0)	33.1 (20.5–102.0)	0.057
NLR/Alb	1.0 (0.4–4.3)	2.3 (0.3–6.7)	0.0327

## Discussion

In this study, we analyzed real-world data from patients with terminal gastric cancer who received end-of-life care at home. Our results identified wide variation in OS, with some patients surviving for only a short time. In addition, the analysis of the short- and long-term OS groups demonstrated an association with serum Alb levels and NLR/Alb. Therefore, serum Alb levels and blood cell count may be useful in predicting the prognosis of patients with end-stage gastric cancer treated at home.

In Japan, approximately 50,000 people die from gastric cancer annually, making it the second leading cause of cancer-related death.^3^ Despite this prevalence, the prognosis for patients in their homes remains uncertain. This discrepancy highlights the gap between the high incidence of gastric cancer-related mortality and the underutilization of home care as an end-of-life option. In this study, one patient survived for up to 406 days, whereas another had a home care period of 0 days. The study suggested that short-term survival may negatively impact trust in home care and increase anxiety in patients and families,^6^ highlighting the importance of accurate prognostication to improve the patient-provider relationship. Traditional Japanese homes often have many steps and different levels, hence the installation of handrails or complete home renovation is needed to make the homes accessible for people undergoing end-of-life care to live comfortably at home. However, such home modifications required in the transition to home care often take time. Even in cases where administrative support is available, patients with an extremely short prognosis may not be able to fully benefit from these modifications. In addition, as Japan experiences both an aging society and an increase in nuclear families, providing end-of-life care at home requires not only home visits by doctors and nurses but also the involvement of caseworkers and other administrative support. Establishing a multidisciplinary care network takes time, hence accurate prognosis prediction for terminal patients is crucial for effective team building and coordination. Therefore, in this study, we focused on the prognosis of patients with terminal gastric cancer who received end-of-life care at home and analyzed patient demographics and blood examination data.

In the present study, prognosis was not related to age (84 vs. 72 years, *p* = 0.070). While some studies suggest that younger patients with gastric cancer tend to have poorly differentiated and advanced stages,^11^ others indicate that age alone does not significantly affect prognosis after curative resection.^12^ Comorbidities, such as hypertension, were more frequent in the OS-L group (25.9% vs. 11.1%, *p* = 0.040). Further studies with larger sample sizes are required to confirm these associations. Furthermore, patients who exhibited a more severe reduction in oral intake at the outset of the visit had a worse prognosis. Oral intake was identified as an important prognostic factor in this study and was also used for PPI.^13^ It has been demonstrated that patients with terminal cancer are capable of ingesting minute quantities orally until the time of death.^8^ Therefore, changes in oral intake may be useful for predicting the prognosis of patients with terminal gastric cancer who are receiving end-of-life care at home.

HOT and strong opioids are key components of end-of-life care for patients with terminal cancer, addressing symptoms such as pain and respiratory distress. In this study, HOT was used in 33.3% of the patients, which is notably higher than the <10% reported in prior studies on patients with terminal cancer in home care settings.^14^ HOT has previously been associated with conditions such as lung cancer, poor performance status, and ascites as risk factors for dyspnea in patients with terminal cancer.^15^ With regard to opioids, their use in 85.2% of the cases in this study is in-line with previous reports showing opioid administration in approximately 88% of the patients with terminal cancer receiving hospital care.^16^ Morita et al. also found that opioid requirements are influenced by factors such as bone metastases, patient age, and brain metastases, all of which must be considered in the home care setting.^16^ Sumimoto et al. identified risk factors for terminal cancer patients with refractory cancer pain as younger age, respiratory cancer, and a history of opioid switches.^17^ In our study, fentanyl transdermal was the treatment of choice in many cases (59.3%). This was thought to be due to the difficulty of oral intake caused by end-stage gastric cancer. Ultimately, health care providers providing home care must have a nuanced understanding of each patient’s clinical presentation to make informed decisions regarding the use of opioids and HOT. Individualized treatment plans that address a patient’s unique symptoms and comorbidities will increase the effectiveness of palliative care and improve the overall quality-of-life of patients with terminal cancer in home settings.

This study observed a statistically significant difference in serum Alb levels between the OS-L and OS-S groups. While previous studies have highlighted indices such as the PNI and NLR as prognostic factors for cancer,^6,7^ no differences were observed in our study. Serum Alb, a commonly used nutritional marker, is a simple and easily understood indicator that may be valuable in clinical practice. Low serum Alb levels have been associated with poor outcomes, and larger studies report an increased risk of death when serum Alb falls <4.2 g/dL at diagnosis.^18^ In patients with stage III/IV gastric cancer, serum Alb levels were significantly lower than in patients with early-stage disease, with a reported cutoff of 4.0 g/dL.^19^ This suggests that serum Alb reflects the progression of gastric cancer and may be useful in predicting the prognosis of patients receiving end-of-life care at home. In this study, NLR/Alb was also a poor prognostic factor in patients with end-stage gastric cancer who were receiving end-of-life care at home. NLR/Alb, as a combined immune status and nutritional index, can minimize the potential bias related to the nutritional and immune statuses of patients with cancer.^5^ Although it has been reported as a prognostic factor for gastric cancer after curative resection,^5^ there are no reports yet regarding its use in the prognostic prediction of patients with terminal gastric cancer receiving end-of-life care. The PNI, calculated using Alb and lymphocyte counts, has been identified as a valuable prognostic indicator in previous studies.^8^ Kanda et al. reported PNI with a cutoff value of 45 and its association with overall survival in pancreatic cancer. Oyama et al. also reported that the shorter the prognosis, the smaller the cutoff value for PNI.^4^ These findings may be due to the progression of gastric cancer, which lowers the Alb level and lymphocyte count, leading to changes in PNI. In our study, the median PNI was 35.5, suggesting that home care physicians should understand that the optimal value of PNI varies with the progression of gastric cancer. However, the current study found no prognostic relevance in this context. Further investigations with larger sample sizes are necessary to confirm the prognostic value of these indicators in end-of-life care at home.

This study has some limitations, including its retrospective nature and small sample size from two institutions, which limit the statistical analysis. We would like to further increase the number of home clinics and increase the number of cases in the future. In addition, missing values in blood examination data limit a comprehensive evaluation. In the present study, information on histology was missing for 55.5% of the cases and could not be studied. Hospital-based clinicians must provide complete data to accurately predict the prognosis, and home-based clinicians should consider performing blood tests when insufficient data are available.

## Conclusions

Our findings suggest that oral intake and serum Alb and blood cell counts may be valuable prognostic markers for predicting outcomes in patients with terminal gastric cancer receiving end-of-life care at home. It is essential to monitor the nutritional status for the management of end-of-life care for patients with gastric cancer at home, which can help improve care strategies for terminally ill patients.

## Data Availability

All data generated or analyzed in this study have been included in the published article. Further inquiries can be directed to the corresponding authors.

## Abbreviations Used

AUC: Area under the curve
Alb: Albumin
HOT: Home oxygen therapy
NLR: Neutrophil-to-lymphocyte ratio
OS: Overall survival
OS-L: long OS
OS-S: short OS
PNI: Prognostic Nutritional Index
PPI: Palliative Prognostic Index
